# The Emotional Landscape of Multiple System Atrophy: A Preliminary Personality-Based Perspective

**DOI:** 10.3390/jcm14196961

**Published:** 2025-10-01

**Authors:** Eleonora Zirone, Giulia Franco, Federica Arienti, Roberta Ferrucci, Alessandro Di Maio, Giacomo Comi, Filippo Cogiamanian, Alessio Di Fonzo, Francesca Mameli

**Affiliations:** 1 Foundation IRCCS Ca’ Granda Ospedale Maggiore Policlinico, 20122 Milan, Italy; eleonora.zirone@policlinico.mi.it (E.Z.); giulia.franco@policlinico.mi.it (G.F.); roberta.ferrucci@unimi.it (R.F.); giacomo.comi@policlinico.mi.it (G.C.); filippo.cogiamanian@policlinico.mi.it (F.C.); alessio.difonzo@policlinico.mi.it (A.D.F.); 2 Hospital S. Anna, 22042 Como, Italy; federica.arienti.med@gmail.com; 3 Department of Oncology and Hemato-Oncology, University of Milan, 20122 Milan, Italy; 4 Department of Pathophysiology and Transplantation (DEPT), Dino Ferrari Centre, University of Milan, 20122 Milan, Italy; alessandro.dimaio@unimi.it

**Keywords:** multiple system atrophy, personality characteristics, Minnesota multiphasic personality inventory-2-restructured form, non-motor symptoms

## Abstract

**Background:** Multiple System Atrophy (MSA) is a rapidly progressing neurodegenerative movement disorder characterized by autonomic failure, parkinsonism, and cerebellar ataxia. While its non-motor symptoms are well-documented, personality features in MSA remain underexplored. This study characterizes the personality traits of non-demented patients with MSA and explores their association with clinical variables. **Methods:** Twenty-six patients with MSA were assessed using the Minnesota Multiphasic Personality Inventory-2-Restructured Form (MMPI-2-RF). Dementia was excluded by Montreal Cognitive Assessment. Descriptive statistics and non-parametric analyses were conducted to examine clinical, demographic, and MMPI-2-RF variables. **Results:** Patients commonly showed elevated scores in somatic domains: Somatic Complaints (39%), Malaise (58%), and Neurological Complaints (85%), as well as in internalizing emotional traits: Low Positive Emotions (39%), Introversion (46%), Suicidal Ideation (46%), and Hopelessness (54%). Externalizing behavioral traits were absent, with only 4–8% of patients showing elevations in aggression or behavioral dysfunction. Strong correlations were found between somatic and emotional traits (r = 0.656, *p* < 0.001), and between Neurological Complaints and disease duration (r = 0.662, *p* < 0.001). **Conclusions:** This exploratory study reveals a distinct personality pattern in MSA, characterized by marked suicidal ideation, emotional vulnerability with internalizing coping, and absence of externalizing behaviors. These features highlight the need for suicide risk screening, interventions to alleviate psychological suffering, and tailored multidisciplinary care. Larger, longitudinal studies are warranted to confirm these preliminary results and clarify whether these traits reflect premorbid personality, early disease manifestations, or secondary responses, as well as their prognostic and clinical relevance.

## 1. Introduction

Multiple System Atrophy (MSA) is a sporadic and rare neurodegenerative movement disorder characterized by a combination of autonomic failure, parkinsonism, and cerebellar ataxia. With an estimated prevalence of 3.4–4.9 cases per 100,000 individuals, MSA is often misdiagnosed due to its overlapping symptoms with Parkinson’s disease (PD) and other atypical parkinsonian disorders [1]. The disease is associated with rapid progression and poor prognosis, with a median survival of 9.8 years from symptom onset [2].

Although MSA is primarily classified into parkinsonian (MSA-P) and cerebellar (MSA-C) subtypes based on the predominant motor manifestations [2], it also encompasses a broad range of non-motor symptoms (NMS) that commonly affect both subtypes, and significantly impair quality of life (QoL) [3].

Alongside severe autonomic failure, the spectrum of NMS in MSA is broad and increasingly recognized as a major determinant of disease burden. Systematic reviews have shown that over 90% of patients experience at least one NMS, with neuropsychiatric disturbances (depression, anxiety, apathy, emotional incontinence), sleep problems (Rapid eye movement sleep behavior disorder, insomnia, excessive daytime sleepiness), cognitive impairment, and fatigue being among the most prevalent. Pain, urinary and sexual dysfunction, as well as gastrointestinal symptoms, also contribute substantially to disability and reduced QoL [1,3,4]. Higher-order cognitive and behavioral changes, including deficits in executive function, attentional control, and social cognition, have been increasingly reported and are thought to reflect the multisystem neurodegeneration of fronto-striatal and limbic circuits [5,6,7,8].

While the impact of NMS is well recognized, only one study to date [9] has explored personality in this population, leaving a gap in understanding their potential contribution to disease burden and patients’ lives. Specifically, Nicoletti et al. [9] focused on obsessive-compulsive personality disorder (OCPD), reporting that OCPD may not be a defining feature of MSA, with a frequency close to healthy controls and significantly lower than those observed in other atypical parkinsonism, such as Progressive Supranuclear Palsy.

Personality traits have already been described in movement disorders, particularly in PD, with consistent evidence supporting the existence of a distinct “parkinsonian personality”, typically marked by industriousness, rigidity, punctuality, cautiousness, and low novelty-seeking [10,11]. Obsessive-compulsive traits are common, particularly in later disease stages, and may be linked to fronto-striatal dysfunction.

Despite the growing recognition of MSA’s complex neuropsychiatric profile, personality in this population remains largely unexplored. This study aims to fill this knowledge gap by evaluating personality traits in non-demented MSA patients using the Minnesota Multiphasic Personality Inventory-2-Restructured Form (MMPI-2-RF), and by investigating their psychological relevance and clinical associations.

## 2. Materials and Methods

A cohort of 26 patients with MSA (12 female, mean years ± SD: age 60.6 ± 5.7, disease duration 3.6 ± 2.1) was enrolled. Participants were recruited during routine neurological evaluations at the Center for Movement Disorders of the Foundation IRCCS Ca’ Granda Ospedale Maggiore Policlinico in Milan and through the patient registry of the MSA Italian Onlus.

Personality was assessed using the MMPI-2-RF, a 338 true/false items self-report questionnaire evaluating a broad spectrum of psychopathological symptoms and maladaptive personality characteristics that includes validity and substantive scales [12]. Responses were collected using dedicated MMPI-2-RF administration and scoring software (Giunti-Testing v: 0.51.7, powered by Giunti Psychometrics, Florence, Italy).

Inclusion criteria were as follows: (i) a diagnosis of MSA according to current consensus criteria [13]; (ii) age between 40 and 75 years; and (iii) absence of dementia, as verified by the Montreal Cognitive Assessment [14]. Exclusion criteria were as follows: (i) history of major psychiatric disorders (e.g., schizophrenia, bipolar disorder); (ii) concomitant neurological diseases; (iii) severe systemic diseases that could interfere with study participation; and (iv) inability to reliably complete the personality inventory.

The study was performed according to the Declaration of Helsinki, approved by the local institutional review board (protocol no. 1253_2021), and written informed consent was obtained from each patient.

Descriptive statistics were used to summarize demographic and clinical characteristics; the continuous variables were assessed using mean and standard deviations, and categorical variables were expressed as frequencies and percentages. The Shapiro–Wilk test was applied to determine the normality of data. Additionally, to identify the most clinically relevant personality characteristics, we focused on those MMPI-2-RF scales for which T-scores exceeded the clinical threshold (≥65) in more than 35% of the sample.

Spearman’s correlation coefficients were calculated to explore associations among these scales, as well as between these scales and demographic or clinical variables. Correlations equal to or exceeding the threshold for moderate strength (r ≥ 0.5) were taken into account [15]. Comparisons between data of MSA-P and MSA-C phenotypes were performed using the Mann–Whitney U test. Statistical significance was established at *p* < 0.05, and analyses were run via Jamovi version 2.6 (The jamovi project (2024), Sydney, Australia).

## 3. Results

Twenty-six non-demented patients with MSA completed the MMPI-2-RF with fully valid protocols, as determined by the instrument’s standard validity scale thresholds [16]. Demographic and clinical characteristics are presented in Table 1.

Somatic dysfunctions were commonly reported. Elevated scores were observed for Somatic Complaints (RC1: 39%), Malaise (MLS: 58%), and Neurological Complaints (NUC: 85%).

Emotional dysfunctions were also prevalent, with a high percentage of patients exhibiting notable clinical thresholds on scales assessing Low Positive Emotions (RC2: 39%), Introversion/Low Positive Emotionality-Revised, (INTR_r: 46%), Suicidal/Death Ideation (SUI: 46%), and Helplessness/Hopelessness (HLP: 54%). Notably, no patients exhibited clinically elevated scores on the Stress/Worry (STW) scale.

**Figure 1:**
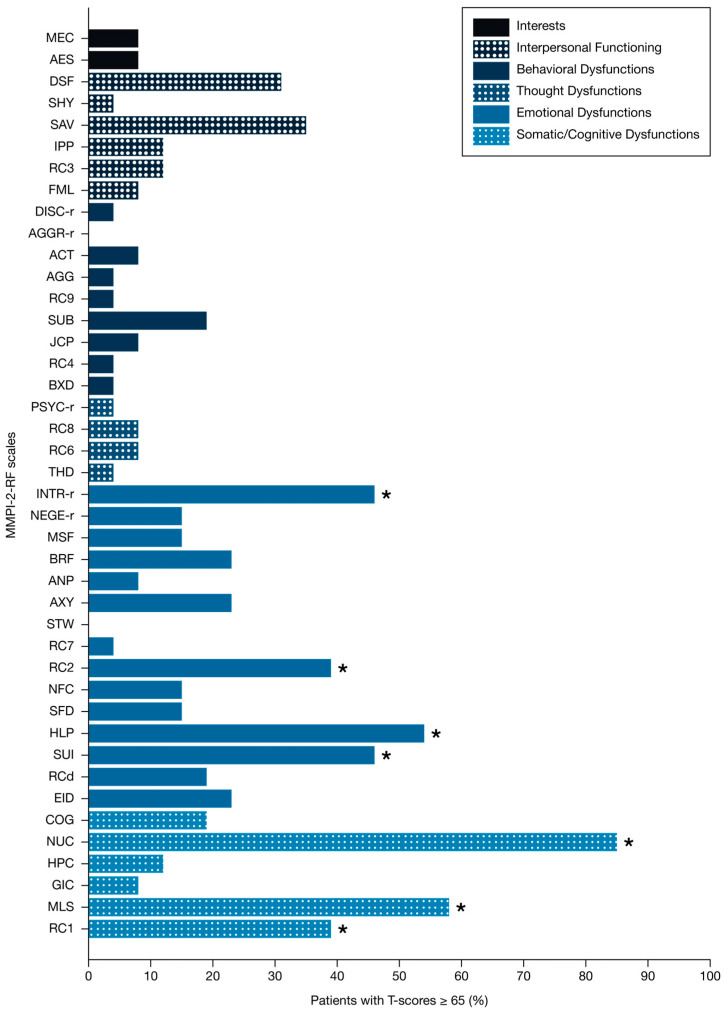
Proportion of patients with Multiple System Atrophy with T-scores ≥ 65 on Minnesota Multiphasic Personality Inventory-2-Restructured Form Substantive Scales. ACT: Activation; AES: Aesthetic-Literary Interests; AGG: Aggression; AGGR-r: Aggressiveness-Revised; ANP: Anger Proneness; AXY: Anxiety; BRF: Behavior-Restricting Fears; BXD: Behavioral/Externalizing Dysfunction; COG: Cognitive Complaints; DISC-r: Disconstraint-Revised; DSF: Disaffiliativeness; EID: Emotional/Internalizing Dysfunction; FML: Family Problems; GIC: Gastrointestinal Complaints; HLP: Helplessness/Hopelessness; HPC: Head Pain Complaints; INTR-r: Introversion/Low Positive Emotionality-Revised; IPP: Interpersonal Passivity; JCP: Juvenile Conduct Problems; MEC: Mechanical-Physical Interests; MLS: Malaise; MMPI-2-RF: Minnesota Multiphasic Personality Inventory-2-Restructured Form; MSF: Multiple Specific Fears; NEGE-r: Negative Emotionality/Neuroticism-Revised; NFC: Inefficacy; NUC: Neurological Complaints; PSYC-r: Psychoticism-Revised; RCd: Demoralization; RC1: Somatic Complaints; RC2: Low Positive Emotions; RC3: Cynicism; RC4: Antisocial Behavior; RC6: Ideas of Persecution; RC7: Dysfunctional Negative Emotions; RC8: Aberrant Experiences; RC9: Hypomanic Activation; SAV: Social Avoidance; SFD: Self-Doubt; SHY: Shyness; STW: Stress/Worry; SUB: Substance Abuse; SUI: Suicidal/Death Ideation; THD: Thought Dysfunction. * MMPI-2-RF scales with >35% of patients scoring T-score ≥ 65.

By contrast, only an extremely small proportion of patients reached significant scores on the behavioural dysfunction scales. Elevation rates were minimal: 0% for Aggressiveness-Revised (AGGR-r); 4% for Aggression (AGG), Antisocial Behavior (RC4), Disconstraint-Revised (DISC-r), Behavioral/Externalizing Dysfunction (BXD); 8% for Activation (ACT) and Juvenile Conduct Problems (JCP) (Figure 1).

Correlation analyses highlighted moderate to strong associations. Low Positive Emotions (RC2) was positively correlated with Introversion/Low Positive Emotionality-Revised (INTR_r: r = 0.825, *p* < 0.001), Helplessness/Hopelessness (HLP: r = 0.503, *p* = 0.009), and Malaise (MLS: r = 0.656, *p* < 0.001). Somatic and Neurological Complaints were strongly interrelated (RC1 and NUC: r = 0.730, *p* < 0.001). Neurological Complaints also showed significant positive correlations with disease duration (r = 0.662, *p* < 0.001).

No statistically significant differences were observed between MSA-P and MSA-C phenotypes across the examined scales (all *p* ≥ 0.05); however, the relatively small sample size precludes drawing definitive conclusions regarding potential subtype-specific differences.

## 4. Discussion

To the best of our knowledge, this is the first study to systematically characterize the personality features of non-demented patients with MSA using the MMPI-2-RF. Our preliminary findings revealed unexpected and psychologically meaningful patterns, suggesting that MSA may be associated with specific personality traits as well as emotional and behavioral characteristics of clinical interest.

Somatic and neurological dysfunctions were commonly reported by the participants, aligning with the multisystemic nature of MSA [1]. Elevated scores on scales assessing neurological complaints and general malaise reflect a pervasive sense of physical debilitation, diminished vitality, and preoccupation with bodily symptoms, findings that are expected, given the clinical burden of the disease. These concerns appear as medical consequences and as central elements of the patients’ psychological experience. The correlation between neurological complaints and disease duration also supports the interpretation that health-related psychological distress in MSA is a meaningful psychological response to progressive neurological disease.

In line with previous evidence [6], emotional difficulties emerged as a substantial component of personality. Elevated scores on key scales indicated a consistent pattern of affective vulnerability, with many patients reporting low positive emotionality and significant anhedonia, suggesting clinically relevant mood deflection. Nearly half of the participants also showed traits of introversion and social withdrawal, as well as suicidal ideation, while markers of hopelessness were present in more than half of the sample. Importantly, a positive correlation was observed between general somatic complaints and affective dimensions, further emphasizing the close interplay between physical symptoms and emotional distress. Notably, however, none of the patients reached clinically significant scores on the Stress/Worry scale. This apparent dissociation between emotional burden and overt tension may suggest an emotionally inhibited or resigned stance, rather than a genuine absence of psychological distress.

Interestingly, the analysis of the behavioural dysfunctions revealed a strikingly uniform pattern of scores within the normative range. This suggests a consistent absence of maladaptive external behaviors across the MSA cohort. Notably, only one patient reached a clinically significant score on the Aggressiveness scale (AGG), further emphasizing the rarity of overtly disinhibited, hostile, or confrontational behaviors in this population. Similarly, the scale measuring heightened levels of excitement (ACT) showed no relevant elevations, remaining well below the thresholds for clinical concern. These findings provide compelling evidence that behavioural dysfunction, such as impulsivity and aggression, are not characteristic of patients with MSA. On the contrary, patients appear to maintain a high degree of behavioral control, which may mask underlying emotional distress, and contribute to the internalized expression of suffering observed elsewhere. Further supporting this pattern, scores on broader domains of behavioral dysregulation and disinhibition remained uniformly within the normative range. In particular, the Behavioral/Externalizing Dysfunction scale (BXD) was elevated in only one patient, whereas approximately 30% of the sample scored in the extremely low range (T-score < 39). This difference suggests heightened behavioral inhibition or restraint. Similarly, no patients reached clinical thresholds on the Aggressiveness-Revised scale (AGGR-r), confirming the lack of overt hostile or confrontational tendencies. Additionally, nearly half of the cohort presented extremely low scores (T-score < 39) on the Disconstraint–Revised scale (DISC-r) assessing impulsivity and behavioral disinhibition. This pattern points to a markedly over-controlled behavioral profile, characterized by high levels of self-regulation and inhibition.

Collectively, our exploratory findings reveal distinct personality traits in patients with MSA, characterized by emotional distress, marked somatic and neurological concerns, and a clear absence of externalizing behaviors. Emotional suffering is expressed through anhedonia, hopelessness, and suicidal ideation, while overt signs of stress are notably absent. Extremely low scores on measures of impulsivity and behavioral disinhibition, along with elevated introversion, point to a passive, inhibited, and over-controlled coping style, marked by emotional withdrawal and social disengagement. This pattern may reflect the involvement of fronto-limbic networks, particularly regions implicated in emotional regulation, behavioral activation, and motivation. The observed inhibition may result from neurodegenerative processes affecting initiative and affective responsiveness, features consistent with the apathy frequently observed in MSA [6].

Compared to other movement disorders, such as PD, where impulsivity and behavioral dysregulation are more prominent, patients with MSA displayed a markedly internalizing emotional style, with reduced positive emotional and low levels of externalizing behavior.

### 4.1. Clinical Implications

The emerging personality pattern provides clinically relevant insights with direct implications for caregiving strategies and patient management.

The absence of overt stress or irritability may lead clinicians to underestimate the depth of patients’ psychological suffering. However, elevated levels of suicidal ideation, hopelessness, and social withdrawal strongly suggest that distress is present, though suppressed or unexpressed. The coexistence of behavioral inhibition with a high burden of internalizing symptoms delineates a particularly concerning profile. Patients at risk of severe emotional suffering may remain silent, invisible to standard clinical observation. This hidden risk can delay recognition of psychiatric comorbidities and limit timely intervention. Moreover, the co-occurrence of elevated suicidal ideation and limited emotional communication may represent a dangerous clinical configuration. In such cases, patients may be unable or unwilling to communicate their suffering effectively, thereby reducing the chances that healthcare providers, caregivers, or family members will detect early warning signs. This highlights the need for heightened vigilance, systematic suicide risk assessment, and proactive screening for internalizing symptoms, even in the absence of overt externalizing behaviors.

From a management perspective, these findings call for a stronger integration of psychological expertise into multidisciplinary care. Clinicians should not rely solely on observable signs of distress but adopt validated screening tools to detect subtle internalizing symptoms and coping vulnerabilities. Psychoeducation for caregivers and healthcare teams may further facilitate recognition of these less visible forms of suffering. Integrating psychological insight into routine care could improve early identification of risk, guide individualized support strategies, and ultimately enhance patients’ QoL. Moreover, recognizing the interplay between somatic burden, disease progression, and emotional vulnerability may foster more holistic care models, in which both motor and non-motor aspects of MSA are addressed as integral parts of the disease process.

### 4.2. Limitations and Future Directions

While the findings of our study offer new perspectives, several methodological considerations should be acknowledged. First, the relatively small sample size reduced statistical power, which may have precluded the detection of more subtle effects, such as phenotype differences or medication confounders, and limits the extent to which these findings can be generalized to the wider MSA population. Nonetheless, the consistency of the observed patterns supports their clinical relevance and warrants replication in larger cohorts. Second, the cross-sectional design does not allow conclusions about the temporal evolution of personality traits, making longitudinal studies particularly important to clarify whether these features represent stable traits, early markers, or changes secondary to disease progression. Third, motor assessments were available only for a subset of patients, which limited the possibility of systematically exploring associations between personality and motor severity; future studies should therefore include comprehensive motor evaluations. Fourth, the exclusion of patients with moderate cognitive impairment, although necessary to ensure the reliability of self-reported data, may have resulted in an underrepresentation of more complex neuropsychiatric profiles, highlighting the need for alternative methods such as informant-based ratings or structured interviews. Finally, reliance on self-report measures may have introduced response biases, especially in individuals with reduced emotional awareness. Although the MMPI-2-RF includes validity scales to mitigate this risk, the integration of multimodal approaches would provide a more complete and accurate characterization of personality features in MSA.

## 5. Conclusions

This exploratory study identifies a distinct personality pattern in MSA, marked by elevated suicidal ideation, emotional vulnerability with internalizing coping styles, and a clear absence of externalizing behaviors. These findings highlight critical clinical needs, including routine suicide risk assessment, targeted interventions to alleviate psychological suffering, and tailored multidisciplinary care strategies. Implementing such integrated neuropsychiatric and psychological management could substantially improve QoL for patients with this progressive disorder.

Future studies with larger cohorts are needed to confirm these preliminary findings and to better delineate the relationship between personality traits and clinical variables in MSA. Longitudinal designs will be particularly valuable to clarify whether the observed personality patterns represent long-standing traits, early manifestations of the disease, or secondary responses to its progression, and to determine their relevance for prognosis and patient care.

## Figures and Tables

**Table 1:** Demographic and clinical features of patients with Multiple System Atrophy.

Demographic and Clinical Features		N (%)
Gender	Female Male	12 (46%) 14 (54%)
Phenotype	MSA-P MSA-C	11 (42%) 15 (58%)
Antidepressant/anxiolytic medication use		6 (23%)
		**mean ± SD**
Age (years)		60.6 ± 5.7
Education (years)		12.0 ± 2.4
Disease duration (years)		3.6 ± 2.1
Adjusted MoCA score		24.3 ± 3.2
LEDD (mg)		262 ± 282
UMSARS I * UMSARS II * UMSARS IV *		24.6 ± 7.0 25.5 ± 7.2 3.0 ± 1.1

LEDD: Levodopa Equivalent Daily Dose; MoCA: Montreal Cognitive Assessment; MSA-C: Multiple System Atrophy-cerebellar; MSA-P: Multiple System Atrophy-parkinsonian; UMSARS: Unified Multiple System Atrophy Rating Scale. * data were collected from ten patients.

## Data Availability

The data presented in this study are available on request from the corresponding author.
